# Editorial of Special Issue “The Self-Assembly and Design of Polyfunctional Nanosystems 2.0”

**DOI:** 10.3390/ijms23084437

**Published:** 2022-04-18

**Authors:** Ruslan Kashapov, Lucia Zakharova

**Affiliations:** A.E. Arbuzov Institute of Organic and Physical Chemistry, FRC Kazan Scientific Center of RAS, 8 Arbuzov Street, 420088 Kazan, Russia

The intention of this Special Issue, entitled “The Self-Assembly and Design of Polyfunctional Nanosystems 2.0”, is to highlight the advantages of spontaneous molecular self-assembly processes in the context of designing new nanomaterials with desired properties. The expected benefit of using this supramolecular approach is a compliance with the main criteria of green chemistry, which is met by the use of an aqueous medium instead of organic solvents and a reduction in synthesis time instead of a large consumption of electricity. In this regard, a supramolecular self-assembly can open up new horizons and knowledge for the development of promising nanoscale compositions and nanotechnological innovations.

Supramolecular assemblies acquire particular relevance in a number of biomedical applications, which is emphasized in both review articles [1,2]. It is fascinating that the biomedical application of supramolecular chemistry is expanding due to the development of antimicrobial aggregates based on peptides and polymers, which have great potential for combating microbial resistance to available antibiotics [1]. In addition, these assemblies can also be useful in water treatment and the production of fabrics and textiles.

In general, the components of nanomaterials based on supramolecular assemblies can be not only organic, but also inorganic or hybrid [2]. At the same time, such compositions are capable of acting as carriers of useful molecules, and the lipid and silicate nanoparticles, modified with a wide range of amphiphilic molecules, are promising transporters. The modification of drug nanocarriers is also being developed through the creation of new hybrid structures consisting of organic and inorganic parts. Such hybrid platforms include cerasomes, which can overcome the limitations inherent in lipid nanoparticles.

Equally fascinating are the vesicles consisting of a single or double layer of block copolymer molecules, called polymersomes, which can be used to encapsulate proteins [3]. Anchoring hydrophobic peptides to membranes significantly enhances protein encapsulation in polymersomes. Cleavage by proteases allows for the more complete removal of protein domains on the surface of vesicles; at the same time, proteases did not affect proteins encapsulated by the inner core of the polymersomes.

The in vitro studies of nanoparticles functionalized with polyamidoamine dendrimers, followed by the conjugation with fragments of polyethylene glycol and folic acid, demonstrated the potential of these dendrimers in the combined chemo- and photothermal therapy of liver cancer [4]. These nanoparticles can efficiently absorb and convert near-infrared light into heat. Doxorubicin encapsulated in the framework of the dendrimer component of nanoparticles is released under the influence of laser radiation, and the combination of chemo- and photothermal therapy further reduces the viability of cancer cells. Supramolecular assemblies can be used to bind not only drugs, but also diagnostic dyes. The authors of [5] report on the development of self-assembling systems based on oppositely charged pillar [5] arenes and surfactants for the encapsulation of 4’,6-diamidino-2-phenylindole, which is used for fluorescent staining of DNA.

The biomedical potential of supramolecular assemblies can be realized in diagnostics. Aqueous polymer solutions can affect the magnetic relaxation behavior of metal complexes used as MRI contrast agents. It was shown in [6] that the addition of polyethyleneimine to aqueous solutions containing gadolinium(III) and citric acid changes the rates of proton NMR relaxation more significantly than polydiallyldimethylammonium chloride. The formation of complexes and associates in the ternary system (gadolinium(III)–citric acid–polyethyleneimine) depends on the pH of the solution. The established patterns of relaxation changes in such systems are very important for the development of prototypes of highly relaxing MRI contrast agents.

An interesting development of achiral liquid crystal polymers for chiral self-recovery is presented in [7], which describes an efficient method for implementing the transfer and preservation of chirality in achiral liquid crystals by combining the strategy of chiral doping and crosslinking. After destruction by UV light, the memorized chiral information in the covalent network made it possible to completely restore the original chiral superstructure. These results meant that a new chiral switch without an additional chiral source could be created from several types of liquid crystal polymers, which could become one of the promising candidates for the fabrication of stimulus-responsive chiro-optical devices.

An analysis of articles published in this Special Issue, entitled “The Self-Assembly and Design of Polyfunctional Nanosystems 2.0”, showed that self-assembly research has great potential for the directed design of nanoscale systems with functional properties. The use of the supramolecular approach to create useful nanomaterials opens up new possibilities for the needs of medicine and biology. Moreover, the basis for the creation of such nanomaterials is mainly polymers of natural and synthetic origin. Thus, the coassembly of polymers serves as a universal strategy for obtaining multifunctional nanoplatforms that can be hierarchically modified with various agents, making it possible to increase their efficiency.
